# Home-based respiratory-gated transcutaneous auricular vagus nerve stimulation for rheumatoid arthritis—a feasibility study

**DOI:** 10.1007/s10067-026-08041-x

**Published:** 2026-03-23

**Authors:** Ankit Parikh, Gwyn Lewis, Hamid GholamHosseini, Kristine Pek Ling Ng, Michal Kluger, Faisal Almesfer, Wee Leong Chang, David Rice

**Affiliations:** 1 School of Clinical Sciences, Auckland University of Technology, Auckland, 0627 New Zealand; 2 Exsurgo Ltd, 45I William Pickering Drive, Rosedale, Auckland, 0632 New Zealand; 3 Department of Physiotherapy, Auckland University of Technology, Auckland, 0627 New Zealand; 4 Department of Electrical and Electronic Engineering, Auckland University of Technology, Auckland, 1010 New Zealand; 5 Department of Rheumatology, Te Whatu Ora - Health New Zealand Waitematā, North Shore Hospital, Shakespeare Road, Takapuna, Auckland, 0622 New Zealand; 6 Department of Anaesthesiology and Perioperative Medicine, Te Whatu Ora - Health New Zealand Waitematā, North Shore Hospital, Shakespeare Road, Takapuna, Auckland, 0622 New Zealand; 7 Department of Anaesthesiology, Faculty of Medicine and Health Sciences, University of Auckland, Auckland, 1010 New Zealand; 8 Department of Biomedical Science, Auckland University of Technology, Auckland, 1010 New Zealand; 9 Health and Rehabilitation Research Institute, Auckland University of Technology, Auckland, 0627 New Zealand; 10 Waitematā Pain Services, Te Whatu Ora - Health New Zealand Waitematā, North Shore Hospital, Shakespeare Road, Takapuna, Auckland, 0622 New Zealand; 11 Institute of Biomedical Technologies, Auckland University of Technology, Auckland, 1010 New Zealand

**Keywords:** RA, Respiratory-gated stimulation, Rheumatoid arthritis, TaVNS, TNF-α, Transcutaneous auricular vagus nerve stimulation

## Abstract

**Introduction/objectives:**

Rheumatoid arthritis is a chronic autoimmune disease that primarily affects the joints, causing pain, inflammation, and long-term joint damage. Transcutaneous auricular vagus nerve stimulation (taVNS) is a promising non-pharmacological intervention for managing pain and inflammation, particularly when delivered in the exhalation phase of respiration. However, until now, the lack of portable equipment has meant the respiratory phase effects have not been evaluated outside of the laboratory or in a home setting. The primary goal of this study was to determine the feasibility of a future clinical trial of home-based respiratory-gated taVNS in people with rheumatoid arthritis.

**Methods:**

This was a single-arm, non-randomised feasibility trial involving 12 individuals with active rheumatoid arthritis. Participation involved 12 respiratory-gated taVNS sessions at home and three laboratory visits for outcome assessments (day 1, day 8, and day 15). Feasibility outcomes focused on recruitment rate, intervention adherence, acceptability questionnaires, and adverse events. In addition, outcome measures related to pain, psychological distress, and key inflammatory biomarkers were assessed.

**Results:**

Feasibility outcomes demonstrated an acceptable recruitment rate, high adherence (90% session completion), excellent usability (9/10), and acceptability (average score 66%, general score 87%), with no severe treatment-emergent adverse events. While single-armed and not powered to determine efficacy, medium to large pre- to post-intervention reductions in pain interference, grip pain intensity, psychological distress, and tumour necrosis factor-alpha levels were observed, with minimal changes in other outcomes.

**Conclusion:**

An adequately powered, randomised controlled trial of home-based respiratory-gated taVNS in people with rheumatoid arthritis is feasible and safe.

**Table:** 

**Key Points** • *To the best of our knowledge, this study is the first to evaluate the feasibility of homebased respiratory-gated (RG) transcutaneous auricular vagus nerve stimulation (taVNS) for the treatment of rheumatoid arthritis (RA).* • *Feasibility outcomes demonstrated high adherence and excellent usability ratings with few treatment-emergent adverse events reported.* • *Home-based RG taVNS using the developed system is safe and feasible to evaluate in an adequately powered, controlled trial.*

**Supplementary Information:**

The online version contains supplementary material available at 10.1007/s10067-026-08041-x.

## Introduction

Rheumatoid arthritis (RA), an autoimmune disease, is the most common form of inflammatory arthritis and, if untreated, may lead to joint damage, chronic pain and loss of joint function. Inflammation within the joint contributes to the activation and sensitisation of peripheral nociceptors, resulting in potential neuroplastic changes in both the peripheral and central nervous systems [1]. Pain in RA can also persist in the absence of overt inflammation, which is thought to be driven mainly by central sensitisation, including impaired descending modulation of nociceptive pathways [2].

Transcutaneous auricular vagus nerve stimulation (taVNS) is a potential non-pharmacological method for managing pain. Vagus nerve stimulation activates several brainstem regions involved in descending pain inhibition [3, 4], likely increases the release of endogenous opioids [5], may alter nociceptive processing at a cortical level [6–8], and has anti-inflammatory effects [9, 10]. All these actions have been hypothesised to have an antinociceptive effect [11, 12]. taVNS is also advantageous in terms of clinical feasibility and costs, as devices can be made small enough to be portable, allowing it to be self-administered at home.

One of the first human trials of implantable vagus nerve stimulation (VNS) in patients with active RA [13] reported inhibition of pro-inflammatory cytokine production and attenuation of disease severity. Another study showed that implantable VNS was not only efficacious in reducing disease severity but was also safe and well-tolerated [14]. Based on these findings, a couple of pilot studies [15, 16] investigated the efficacy of tcVNS and taVNS in patients with RA, with results supporting its safety and efficacy in reducing disease activity.

Additionally, activity in the vagal brainstem nuclei is cyclically modulated by respiration [4]. Recent functional magnetic resonance imaging (fMRI) studies [6, 17] have shown that exhalation respiratory-gated (RG) taVNS evoked an fMRI signal increase in brain regions involved in descending pain modulation. Hence, RG taVNS, synchronised with the exhalation phase of the respiration cycle, may improve its potential for pain reduction.

The outcomes of a previous study [18] contributed to the development of a prototype RG taVNS system that is compatible with home use. While several taVNS devices are commercially available [19–21], none of them is RG. To the best of our knowledge, there are no prior studies evaluating unsupervised, remote RG taVNS in people with RA. Hence, we needed to explore key feasibility measures before designing a clinical trial. The primary objective of this study was to assess the feasibility of a future clinical trial of the RG taVNS intervention using the prototype device in individuals with RA. Feasibility was assessed in terms of participant recruitment, adherence to treatment, safety, usability, and acceptability. Secondary objectives were to estimate changes from pre- to post-intervention in pain, psychological distress, disability, and inflammation, and to explore possible dose-response relationships in these outcomes.

## Materials and methods

### Study population

A sample size of 12 was chosen based on the number of participants that were considered feasible to recruit over 6 months, rather than any formal power calculation.

#### Location

The study was conducted at a university laboratory located at North Shore Hospital, Auckland, New Zealand.

#### Recruitment criteria

Participants were recruited from the Rheumatology Outpatient Department of North Shore Hospital. Individuals who were aged 18 years and over, diagnosed with adult-onset RA according to the American College of Rheumatology and the European League Against Rheumatism 2010 RA classification criteria [22], and had the presence of at least 3/28 swollen and/or at least 3/28 tender joints, with one tender joint being in the hand or wrists, were eligible for participation. Key exclusion criteria included current ear infection, previous vagotomy, currently implanted electrical or neurostimulation device, and changes in or unstable dosing regimens for disease-modifying drugs in the last 4 weeks. A full list of inclusion and exclusion criteria can be found in the supplementary material.

### Study design

This study was an open-label, single-arm, repeated-measures feasibility study conducted over a period of 2 weeks. Participants underwent 14 sessions of RG taVNS, with two sessions supervised at the local hospital and 12 sessions completed independently at home. A single-arm design was chosen to assess the fundamental feasibility measures related to the safety and delivery of the new intervention before comparative evaluation.

Participants visited the hospital for three assessment sessions on day 1, day 8, and day 15. During these visits, self-report questionnaires and quantitative sensory testing were completed, and blood samples were collected to evaluate inflammatory biomarkers. Additionally, the first two hospital visits included a supervised 20-min RG taVNS session, during which participants were trained on the use of the equipment in the kit.

For the home-based sessions, participants were instructed to complete one 20-min RG taVNS session daily for 12 days. Adherence was monitored through a mobile application that automatically recorded session completion data.

### RG taVNS

The RG taVNS kit included a prototype system consisting of a fingertip pulse sensor, a respiration sensor strap, a commercial taVNS (Vagustim, Turkey) device, a custom auricular electrode housed in a headband, alcohol wipes, and a Samsung Galaxy Tab A7 with Android operating system version 12 running the mobile application. As part of the induction during the first assessment session, participants were trained on how to insert and position the stimulation electrode on the cymba concha region of the left ear, how to position the fingertip heart rate sensor and respiration strap, and how to use the mobile application. If the participants had poor hand dexterity, they were asked to be accompanied by a person who could assist them in fitting the equipment during the home sessions.

**Fig. 1:**
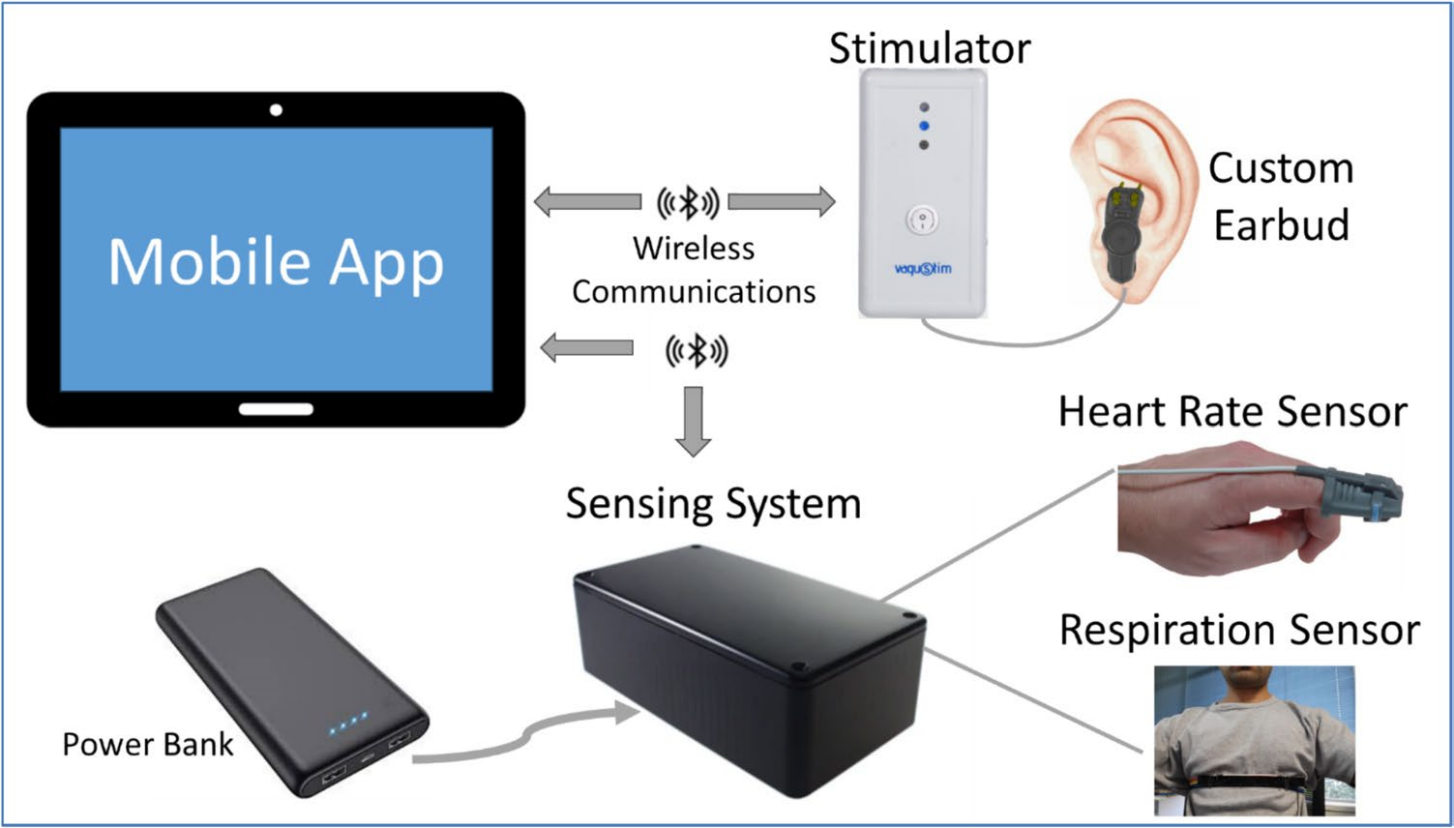
Participant kit containing a 10-in. mobile tablet, a Vagustim device with a custom left ear electrode, a sensor box with connections to a fingertip sensor, a respiration sensor, and a power bank

taVNS was delivered via symmetrical biphasic rectangular waveforms at 30 Hz frequency [4, 23, 24] with a 1 ms pulse width [25, 26] for 1 s during exhalation [17, 27–29]. Stimulation was delivered within 500 ms of the onset of exhalation. Participants were instructed to use the mobile application’s device setup screen to set a stimulation intensity (constant current between 1 and 5 mA in 0.1 mA increments) that felt strong but not uncomfortable [27–29]. The application stored each user’s stimulation intensity, starting subsequent sessions at 5 points below the previous level. Participants then adjusted it to match the initial instructions. On the setup screen, visual feedback from respiration and fingertip heart rate sensors was shown. The application verified sensor data before allowing the user to proceed. After setup, a 2-min baseline was recorded to calibrate exhalation detection using respiration data. Then, the 20-min stimulation session began, during which users could pause and adjust intensity. The application instructed users to sit comfortably, relax, and close their eyes if needed. A timer displayed the remaining time, and audio prompts every 5 min updated progress. Optionally, the users could also play preloaded ambient music. After the first in-person assessment session, participants carried the kit home. Figure 1 shows the components of the kit.

**Fig. 2:**
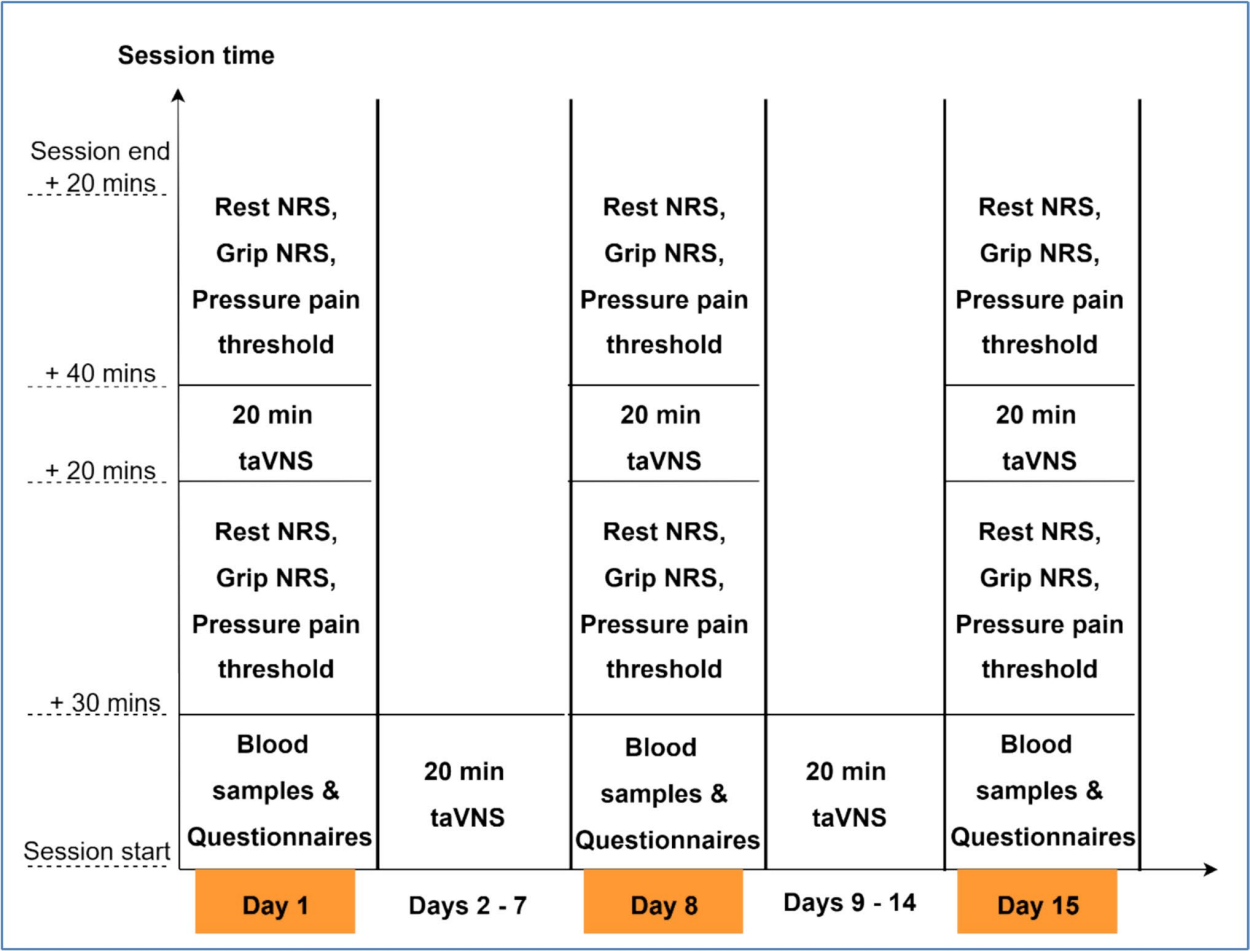
Study protocol for the 2 weeks, outlining the flow of events during the three hospital visits. Note: NRS, numerical rating scale from 0 to 10

Participants were provided with the researcher’s contact details if technical support or other assistance was required. If participants missed a single session, a text message and email were sent reminding them to complete the next session as planned. If participants failed to complete two consecutive sessions, the coordinating investigator made a phone call to encourage adherence and troubleshoot any issues with the training. Figure 2 illustrates the timeline of the study protocol for the 2 weeks.

### Protocol deviation

Due to a procedural error, we failed to record heart rate variability data via the fingertip sensor pre- and post-RG taVNS during the hospital visits. This error resulted in a lack of data needed to report changes in resting root mean square of successive R-R interval differences (RMSSD) and correlations between within-session changes in RMSSD and changes in pain measures (resting pain, grip pain, and pressure pain thresholds), as we originally planned.

## Outcome measures

### Feasibility measures

#### Eligibility and recruitment

A research nurse maintained a log documenting the number of people with RA who were screened, ineligible, eligible, consented, or declined. Feasibility outcomes were reported on:

- The proportion of screened people with RA who met the inclusion criteria as a percentage of the total screened people (with a success criterion of 30% or above).
- The percentage of eligible participants who consented to participate in the study (with a success criterion of 60% or above).

#### Adherence

The mobile application recorded the number of taVNS sessions started and completed. Adherence was reported as a percentage of the total possible at-home sessions (out of 12 per participant) completed before the final assessment, with a success criterion of 75% or above.

#### Usability and acceptability

At the end of the final assessment session (day 15), participants were asked to complete usability and acceptability questionnaires. The usability questionnaire asked participants to rate each question on a scale of 1 (Strongly Disagree) to 10 (Strongly Agree). The questions were based on guidelines provided by a systematic review of e-health applications [30] and a study evaluating the usability of mobile health applications among participants with chronic health conditions [31]. The acceptability questionnaire was based on the theoretical framework of acceptability [32] for healthcare interventions. The responses to this questionnaire were rated on a scale of 1 to 5, with each question containing an explanation of its corresponding scale. Individual items and total scores from both questionnaires were evaluated. An overall acceptability score of 60% or higher was considered adequate, given the prototype design.

#### Adverse events

Any participant withdrawals, reasons for withdrawal and any treatment-emergent adverse event (TEAE) during the study period were reported and documented. TEAEs were assessed during the day 8 and day 15 visits. A TEAE was considered any unfavourable and unintended sign, symptom, or disease that occurred during the study period, which may or may not be considered related to the study.

### Pre- to post-intervention effects

Pre- to post-intervention effects were explored through assessments at days 1, 8, and 15 of pain intensity (resting numerical rating scale [NRS], gripping NRS), pain sensitivity (pressure pain threshold [PPT]), pain interference (Brief Pain Inventory [BPI]), psychological distress (Depression, Anxiety and Stress Scale [DASS-21]), physical function (Health Assessment Questionnaire-Disability Index [HAQ-DI]), and blood biomarkers (C-reactive protein [CRP], tumour necrosis factor-α [TNF-α], interleukin [IL]−1β, IL-10, IL-17A). Missing data were imputed using the last observation carried forward.

The data collection protocols for each session are shown in Fig. 2. Before and immediately after the 20-min stimulation at each visit, pain at rest and grip pain (5 repetitions of a 5 kg grip) were assessed using a 0 (No Pain) to 10 (Worst Pain Possible) NRS and PPTs were assessed. PPT was assessed using an algometer (SBmedic, Sweden) at two standardised sites, the wrist (at a point behind the ulnar styloid) of the dominant hand and the contralateral tibialis anterior, at a ramping rate of 30 kPa/s. To assess PPT at each location, the pressure was increased until the participant reported the onset of pain, at which point pressure was ceased and recorded (kPa). A mean of three measurements was taken at each site, with a 30-s rest between repetitions.

Venous blood samples were collected at the start of each visit for blood biomarker analysis. CRP measurements were completed on the same day at the laboratory located in the hospital. For post-study cytokine analysis, plasma was separated by centrifugation and aliquoted into four 1-mL vials, which were stored at −80 °C until analysis. Cytokine measurements were performed at the AUT-Roche Diagnostics Laboratory (Auckland, New Zealand) using Abcam enzyme-linked immunosorbent assay (ELISA) kits (Abcam, UK) according to the manufacturer’s protocol. Absorbance was measured at the appropriate wavelength using a Thermo Fisher microplate reader (Thermo Fisher Scientific, USA).

## Data analysis

Effect sizes were calculated from baseline to mid-intervention (day 8) and baseline to post-intervention (day 15) for resting pain, grip pain, PPTs, BPI interference score, DASS-21 total score, HAQ-DI, and inflammatory biomarkers (CRP, TNF-α, IL-1β, IL-10, and IL-17A). Inflammatory biomarkers measured below the limit of detection (LOD) were substituted with a value of “LOD/√2” [15]. If more than 10% of the data were below LOD, the biomarkers were excluded from the analysis. The effect sizes were calculated using Cohen’s *d*_*z*_ [33] (Eq. 1), which represents the standardised mean difference effect size for within-subjects measurements. The effect sizes were interpreted as small (0.20–0.49), medium (0.50–0.79), and large (≥ 0.80).

1 $$Cohen^\prime{s}\;dz=\frac{Mdiff}{Sdiff}$$

Note: *M*_diff_ is the mean of the differences between the two measurements. *S*_diff_ is the standard deviation of the difference.

## Results

### Participant characteristics

Twelve participants, with a mean age of 62 years (range, 33–82; 10 female), were recruited for the study (Table 1). Eleven participants completed the study. One participant (8%) withdrew from the study after completing the second assessment session (day 8), as they felt the daily 20-min intervention sessions were too long.

**Table 1:** Summary of participant characteristics

	*N* = 12
**General characteristic**	
Age (years)	62 (13)
Female	10 (83%)
Weight (kg)	80 (23)
Height (cm)	163 (11)
BMI (kg/m^2^)	30 (8)
Ethnicity	
European	9 (75%)
African	1 (8%)
Asian	2 (17%)
**Medications**	
Disease modifying antirheumatic drugs	
Methotrexate	4 (25%)
Sulfasalazine	5 (25%)
Hydroxychloroquine	2 (17%)
Leflunomide	2 (17%)
Rituximab	2 (17%)
Etanercept	1 (8%)
Corticosteroids	
Prednisone	3 (25%)
Analgesics	
Paracetamol	2 (17%)
Meloxicam	1 (8%)

Data are mean (± 1 standard deviation) or number (proportion), *BMI* body mass index

### Feasibility measures

#### Recruitment

**Fig. 3:**
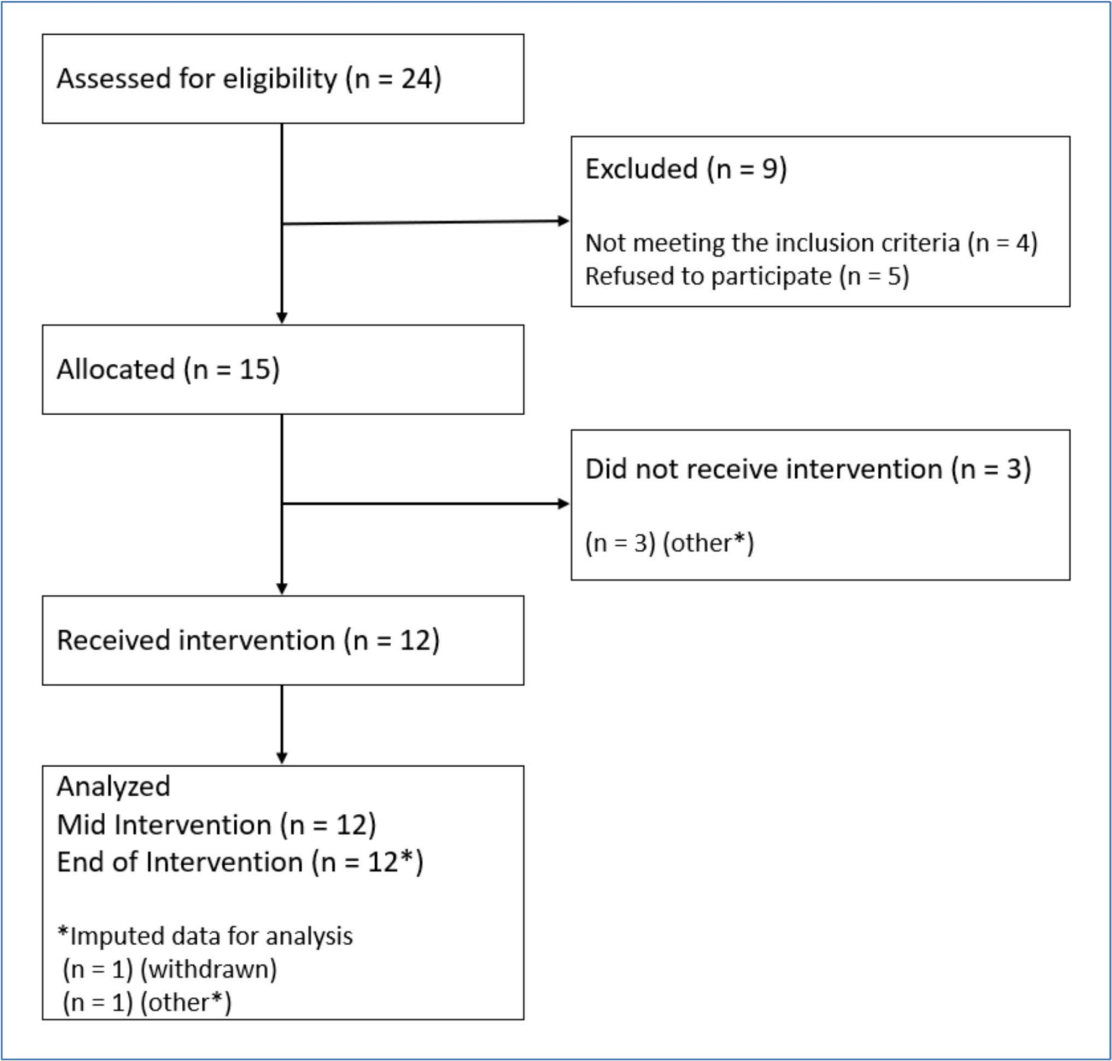
CONSORT flow diagram for the study. Note: *Other, other reasons, for example, being unable to find time or unexpected changes in personal circumstances

Between 12 August 2024 and 12 February 2025, a total of 24 participants were screened (Fig. 3). Twenty (83%) of the screened participants were eligible, exceeding the feasibility criterion of 30%. Of these, 15 (75%) agreed to participate, and 12 (60%) were successfully recruited into the study (Fig. 3), meeting our feasibility criterion of 60%. Two participants who agreed to participate were not recruited as they could not wait until the equipment was available, and one participant did not respond to the recruitment follow-up.

#### Adherence

Participants completed 90% of the total possible at-home sessions, surpassing our feasibility criterion of 75%. Overall, participants started 132 sessions at home out of a possible 144 sessions. Of these, 130 sessions were completed, with two participants unable to complete one session each due to technical difficulties. A total of two reminders were sent throughout the study, one to each of two participants who had missed a session.

#### Usability and acceptance

The average usability rating score was 9 out of 10. The average acceptability score was 66%, with a general acceptability of 87%. Overall, the acceptability score met our feasibility criterion of 60%.

Five participants requested technical assistance on six occasions. In two cases, participants had forgotten their mobile application login PIN code. In two instances, the in-ear electrode wires were damaged. The issue resulted in the participants missing a session and was resolved by providing them with a replacement the next day. One participant requested technical assistance with noisy fingertip-based sensor data during the session. This issue was resolved by the participant repositioning their hand. The last technical assistance issue related to the respiration sensor being unable to detect exhalation for one participant. The issue was resolved by repositioning the sensor strap.

#### Adverse events

Six (50%) participants reported a total of 14 TEAEs during the study (Table 2). None of these was classified as a severe event (grade 3 or higher), and only two events were related or possibly related to the intervention. Of these, the ear rash was resolved by ceasing the use of the supplied alcohol wipes. Vertigo reported by the same participant was considered possibly related to the intervention.

**Table 2:** Treatment-emergent adverse events (TEAEs), by the Common Terminology Criteria for Adverse Events (CTCAE v.5)

Participant	Type of TEAE	CTCAE grade
P001	Arthritis	2
P001	Headache	2
P001	Headache	2
P003	Sore throat	1
P004	Hypertension	2
P004	Arthritis	1
P004	Bone pain	1
P004	Bone pain	1
P005	Diarrhoea	1
P005	Hyperuricemia	2
P007	Palmar-plantar erythrodysesthesia syndrome (skin rash on the left auricle)	1
P007	Vertigo	2
P010	Headache	1
P010	Pain	1
**Not related**		**12**
**Possibly related**		**1**
**Related**		**1**
**Withdrew due to TEAEs**		**0**
**Total TEAEs**		**14**

### Pre- to post-intervention changes in outcomes

Table 3 shows the effect sizes calculated from baseline to mid-intervention and from baseline to the end of the intervention. Data from all participants (*N* = 12) are included in this table. For the end of the intervention, data from two participants were imputed by carrying forward their values from mid-intervention. These exceptions include one participant who withdrew after the second assessment session and one participant who, in the second week of their intervention, had an episode of gout following the consumption of seafood, which led to intense pain and affected their outcomes.

**Table 3:** Mean effects from baseline to mid-intervention, and from baseline to the end of the intervention

Outcome measures	Baseline mean (± SD)	Mid intervention		End of intervention	
		Week 1 mean (± SD)	Cohen’s ***dz***	Week 2 mean (± SD)	Cohen’s ***dz***
BPI interference (0–10)	4.8 (± 2.4)	3.1 (± 3.0)	**0.68**	3.1 (± 2.7)	**0.83**
DASS-21 (0–42)	41.3 (± 28.6)	28.0 (± 21.8)	**1.13**	21.8 (± 20.1)	**1.66**
HAQ-DI (0–3)	1.1 (± 0.7)	0.9 (± 0.9)	**0.53**	1.0 (± 0.9)	0.27
Rest pain (0–10)	4.7 (± 2.3)	3.3 (± 2.2)	**0.57**	3.6 (± 2.2)	0.47
Grip pain (0–10)	5.3 (± 2.1)	4.0 (± 2.8)	**0.64**	3.4 (± 2.2)	**1.12**
PPT wrist, kPa	210.3 (± 91.1)	209.0 (± 102.1)	0.01	195.8 (± 98.0)	0.21
PPT leg, kPa	227.8 (± 95.5)	224.4 (± 102.6)	0.04	200.8 (± 105.7)	0.35
CRP, mg/L	10.8 (± 9.1)	11.5 (± 11.6)	−0.12	12.0 (± 10.9)	−0.24
TNF-α, pg/mL	71.6 (± 51.9)	46.9 (± 12.3)	**0.50**	53.6 (± 16.1)	0.34
IL-10, pg/mL	556.0 (± 770.1)	689.7 (± 807.9)	−0.33	459.5 (± 618.7)	**0.51**

Values in bold highlight medium and large effects. Positive effect sizes relate to decreases in measurements compared to baseline. IL-1β and IL-17A were analysed but not included in this table, owing to more than 10% of the data being under the limits of detection. SD, standard deviation; BPI, Brief Pain Inventory; DASS-21, Depression Anxiety and Stress Scale short form; HAQ-DI, Health Assessment Questionnaire- —Disability Index; PPT, pressure pain threshold; CRP, C-reactive protein; TNF-α, tumour necrosis factor-α; IL, interleukin; kPa, kilopascal; mg/L, milligrams per litre; pg/mL,picograms per millilitre

## Discussion

To the best of our knowledge, this study is the first to evaluate home-based RG taVNS. The primary aim was to assess the feasibility of a future clinical trial of home-based RG taVNS in people with RA. Findings from the recruitment metrics, safety, adherence, and usability/acceptability suggest a future clinical trial is feasible.

The recruitment rate of 60% was comparable to other pilot/feasibility non-invasive VNS studies in people with RA [15, 16]. Notably, two participants who consented were not recruited due to equipment unavailability, suggesting that with sufficient resourcing, this would not be a barrier in a future clinical trial. The majority of the screened (75%) and recruited (83%) participants were females, consistent with global epidemiological data highlighting a higher burden of RA in females [34].

There were two instances of minor, transient TEAEs that were related or possibly related to the study, and none of the participants withdrew due to TEAEs or reported any severe TEAEs, indicating acceptable safety. While the TEAE of skin rash or skin irritation is consistent with previous studies [35, 36], in our case, the participant reported resolution after discontinuing the use of the alcohol wipe to clean the ear, suggesting skin irritation due to the electrode interface may not have been the primary issue. The other TEAE of vertigo has been referred to as dizziness in the recent analysis of side effects of taVNS [37]. However, unlike another study [38], the participant did not have to stop the daily sessions temporarily, and the issue resolved on its own.

Adherence (90%) matched those observed in recent home-based taVNS studies [39, 40], which ranged from 66 to 90%. Being a respiratory-gated study that required additional sensor setup, relatively high adherence could be attributed to the user-friendly mobile application and a relatively short 20-min session length compared to the 60-min sessions in the above-mentioned home-based studies. Adherence could be further improved in future studies by allowing participants to perform shorter sessions, as one participant withdrew from the study for this reason. Sending reminders to participants who missed a session could also have increased adherence. However, as it only occurred on two occasions, it did not have a large impact.

The participants rated their ability to set up the equipment, feel stimulation throughout the session, and the ease and comfort of setting up and wearing the equipment with an average score of 9 out of 10, suggesting that the equipment and application were highly usable. All participants successfully wore and positioned the respiration sensor correctly during the first visit. One participant had challenges positioning the respiration sensor to detect changes in respiration, as they were habitual chest breathers. This issue was resolved by repositioning the sensor on their chest rather than their abdomen. Two instances of accidental in-ear electrode damage suggest that future electrode design should incorporate improved wire stress management with robust bend protection and cable routing to reduce tension on the connections. Together with the high adherence, these findings suggest that, with minor modifications, the equipment is suited for home use.

Average responses to acceptability questions ranged from 2.75 to 4.33 out of 5. Participants rated their affective attitude towards the study, perceived effectiveness, intervention coherence, and ability to participate in future studies with an average score of 4 out of 5. The questions regarding the effort required to participate and the costs associated with engagement in such a study were scored lower. One reason for these low scores was the requirement of three 2-h hospital visits over 2 weeks during business hours. While these responses demonstrate the effort of participating in a research study, they may not reflect the effort to participate and the costs of engagement in home-based RG taVNS more broadly. However, future studies could consider mitigating the impacts of these factors by reducing the number of visits, shortening the duration of visits, offering schedules outside business hours, or offering at-home visits.

Being a single-arm study, the ability to draw inferences regarding the causality of RG taVNS on any of the pre- to post-intervention effects observed is impossible. However, changes were largely in the expected direction. The reductions in pain interference, psychological distress and grip pain between baseline and the end of week 2 reflect a large effect size, while the reductions in resting pain and TNF-α at mid-intervention were medium. Our findings are largely consistent with the small reductions in HAQ-DI and a slight increase in CRP observed in a similar pilot study of taVNS [16]. Contrary to our findings, Drewes et al. [15], who used tcVNS, observed a reduction in CRP, but only in their high disease activity group. However, we observed larger reductions in TNF-α. These blood biomarker changes also corroborate with a recent systematic review and meta-analysis of VNS on inflammation [41], which noted consistent changes in TNF-α only. Reductions in TNF-α in response to taVNS are possible through vagally mediated activation of the cholinergic anti-inflammatory pathway and the hypothalamic-pituitary-adrenal axis pathway, leading to an anti-inflammatory response [42]. Future controlled studies are needed to determine the specific contributions of RG taVNS, as complex time-dependent fluctuations in cytokines have been observed in other studies in RA [13].

In July 2025, the US Food and Drug Administration approved Setpoint Medical’s (Valencia, CA) implantable VNS device for RA after their recent randomised controlled trial [43]. This is a promising development, despite the invasive procedure having higher cost implications and carrying surgical risks. While there is a recent randomised controlled trial for taVNS in the RA population [44], it primarily looked at changes in RA disease activity, which we did not. Their findings suggest that taVNS did not meaningfully improve RA disease activity. However, they used a stimulation frequency of 20 kHz, which is much higher than the usual 10 to 100 Hz range and may block conduction in, rather than stimulate vagal nerve afferent fibres [45].

Overall, the larger effect sizes for grip pain, pain interference, and psychological distress after 2 weeks of treatment, as well as sustained, albeit lesser effects on TNF-α in the second week, suggest that future controlled studies should consider a longer intervention period (e.g. 4–6 weeks) with regular assessment of the outcome measures to evaluate changes in effects over time.

### Strengths and limitations

This is the first study to evaluate the feasibility of a home-based RG taVNS intervention. A custom in-ear electrode was developed that targeted the cymba concha region of the auricle and did not require the use of conductive gel for effective stimulation. This development was crucial as the cymba concha is primarily innervated by the auricular branch of the vagus nerve.

Several limitations of this study should be noted. Our study design precludes causal inferences regarding the effects of RG taVNS on treatment outcomes. The sample size was relatively small (*n* = 12) and was arbitrarily chosen, based on the number of participants we thought would be feasible to recruit within 6 months. We used the last observation carried forward method to handle missing data, which is not usually considered optimal and can increase bias. However, it is commonly employed in pilot/feasibility studies as an alternative to losing the data completely for the affected participants. The study length of 2 weeks may not be long enough to achieve the full effects of stimulation, as recent home-based taVNS studies have ranged from 4 to 12 weeks.

## Conclusion

This study supports the feasibility and safety of home-based RG taVNS for RA using the portable prototype system. While our single-arm, uncontrolled study design precludes causal inferences, medium to large pre- to post-intervention reductions were observed in pain, pain interference, psychological distress and serum TNF-α. Based on the potential dose–response relationships in some outcomes, future randomised controlled trials of RG taVNS in a RA population may wish to include intervention periods of at least 2 weeks.

## Supplementary Information

Below is the link to the electronic supplementary material.

ESM 1 (PDF 579 KB)

ESM 2 (XLSX 10.2 KB)

ESM 3 (XLSX 19.3 KB)

ESM 4 (DOCX 33.9 KB)

ESM 5 (PDF 601 KB)

ESM 6 (XLSX 11.5 KB)

## Data Availability

Data used for statistical analysis and supporting the conclusions of this study are included in the article and its supplementary material. Access to raw sensor datasets may be limited and could be requested from the corresponding author.
