# Supplementary material for: Home-based respiratory-gated transcutaneous auricular vagus nerve stimulation for rheumatoid arthritis—a feasibility study
Source: Clin Rheumatol. 2026 Mar 23;45(5):2627–38. doi: 10.1007/s10067-026-08041-x (PMC13068677; doi:10.1007/s10067-026-08041-x)
Supplement: Supplementary file 1 — (PDF 579 KB) [file 10067_2026_8041_MOESM1_ESM.pdf]

Respiratory-gated taVNS study acceptability questionnaire

A recently developed theoretical framework of acceptability (TFA) proposes seven component constructs (affective attitude, burden, ethicality, intervention coherence, opportunity costs, perceived effectiveness, and self-efficacy) to help identify characteristics of interventions that may be improved.

[https://bmchealthservres.biomedcentral.com/articles/10.1186/s12913-022-07577-3]

developer@exsurgorehab.com Switch account

Not shared

* Indicates required question

Affective attitude

How an individual feels about the intervention

How comfortable did you feel in participating in this ear stimulation study? *

Very uncomfortable	Uncomfortable	No opinion	Comfortable	Very comfortable
1	2	3	4	5

	1	2	3	4	5	
Very uncomfortable	○	○	○	○	○	Very comfortable

Burden

The amount of effort required to participate in the intervention

How much effort did it take to engage with the ear stimulation study? *

This metric will be scored in reverse

No effort at all	A little effort	No opinion	A lot of effort	Huge effort
1	**2**	**3**	**4**	**5**

	1	2	3	4	5	
No effort at all	○	○	○	○	○	Huge effort

Perceived effectiveness

The extent to which the intervention is perceived to have achieved its objective

Ear stimulation has resulted in lower pain and inflammation *

Strongly disagree	Disagree	No opinion	Agree	Strongly agree
1	**2**	**3**	**4**	**5**

	1	2	3	4	5	
Strongly Disagree	○	○	○	○	○	Strongly Agree

Intervention coherence

The extent to which the participant understands how the intervention works

It is clear to me how ear stimulation will help manage my arthritis. *

Strongly disagree	Disagree	No opinion	Agree	Strongly agree
1	2	3	4	5

	1	2	3	4	5	
Strongly Disagree	○	○	○	○	○	Strongly Agree

Self-efficacy

A participant's confidence that they can perform behaviour(s) required to participate in the intervention

How confident do you feel about engaging with a similar ear stimulation study in future? *

Very unconfident	Unconfident	No opinion	Confident	Very confident
1	2	3	4	5

	1	2	3	4	5	
Very unconfident	○	○	○	○	○	Very confident

Opportunity costs

The benefits, profits or values that would have to be given up to engage with the intervention

This ear stimulation study interfered with my other priorities *

This metric will be scored in reverse

Strongly disagree	Disagree	No opinion	Agree	Strongly agree
1	**2**	**3**	**4**	**5**

	1	2	3	4	5	
Strongly Disagree	○	○	○	○	○	Strongly Agree

General acceptability

The general acceptability item has been included as in some instances, an overall acceptability item may be useful and to allow for researchers to explore which of the 7 TFA constructs influences/ drives participants' general acceptability judgment.

How acceptable was ear stimulation to you? *

Completely unacceptable	Unacceptable	No opinion	Acceptable	Completely acceptable
1	**2**	**3**	**4**	**5**

	1	2	3	4	5	
Completely unacceptable	○	○	○	○	○	Completely acceptable

Participant ID *

Your answer

Submit

Clear form

Never submit passwords through Google Forms.

This content is neither created nor endorsed by Google. - Contact form owner - Terms of Service - Privacy Policy

Does this form look suspicious? Report

Google Forms
