# Supplementary material for: Home-based respiratory-gated transcutaneous auricular vagus nerve stimulation for rheumatoid arthritis—a feasibility study
Source: Clin Rheumatol. 2026 Mar 23;45(5):2627–38. doi: 10.1007/s10067-026-08041-x (PMC13068677; doi:10.1007/s10067-026-08041-x)
Supplement: Supplementary file 4 — (DOCX 33.9 KB) [file 10067_2026_8041_MOESM4_ESM.docx]

Recruitment Criteria

Participants were recruited from the Rheumatology outpatient department of North Shore Hospital. Individuals who were aged 18 years and over, diagnosed with adult-onset RA according to the American College of Rheumatology and the European League Against Rheumatism 2010 RA classification criteria [1] and had the presence of at least 3/28 swollen and/or at least 3/28 tender joints, with one tender joint being in the hand or wrists, were eligible for participation.

A research nurse screened potential participants and deemed them ineligible if they met any of the following criteria:

- Current ear infection (otitis media or otitis externa)
- Poor hand dexterity and no access to assistance with fitting the auricular electrodes and the respiration sensor strap
- Changes in oral or biologic disease-modifying antirheumatic drugs in the last 4 weeks
- Intra-articular or intramuscular corticosteroids within 2 weeks prior to study entry
- Unstable dosing regimen of non-steroidal anti-inflammatory drugs or analgesics in the last 2 weeks
- History of arrhythmia, myocardial infarction in the last 12 months, currently on beta-blocker medication or history of stroke affecting the brainstem
- Previous vagotomy
- Currently implanted electrical and/or neurostimulator device
- Active malignancy or history of active malignancy in the last 2 years, with the exception of non-melanoma skin carcinoma or carcinoma in situ
- Severe comorbidities, which, in the judgement of the study physicians, would impact the safety of study conduct
- Known cognitive impairment
- Psychiatric illness with active psychosis
- Pregnant

References:

[1] J. Kay and K. S. Upchurch, “ACR/EULAR 2010 rheumatoid arthritis classification criteria,” *Rheumatology*, vol. 51, no. suppl_6, pp. vi5–vi9, Dec. 2012, doi: 10.1093/RHEUMATOLOGY/KES279.
