# Supplementary material for: Home-based respiratory-gated transcutaneous auricular vagus nerve stimulation for rheumatoid arthritis—a feasibility study
Source: Clin Rheumatol. 2026 Mar 23;45(5):2627–38. doi: 10.1007/s10067-026-08041-x (PMC13068677; doi:10.1007/s10067-026-08041-x)
Supplement: Supplementary file 5 — (PDF 601 KB) [file 10067_2026_8041_MOESM5_ESM.pdf]

Respiratory-gated taVNS study usability questionnaire

This usability questionnaire is based on the usability criteria specified in the following paper

[https://www.ncbi.nlm.nih.gov/pmc/articles/PMC6241759/]

developer@exsurgorehab.com Switch account

Not shared

* Indicates required question

Learnability

With the following questions we are trying to evaluate the ease of learning the functionality and behaviour of the system

It was easy to learn to use the application. *

	1	2	3	4	5	6	7	8	9	10	
Strongly Disagree	○	○	○	○	○	○	○	○	○	○	Strongly Agree

It was easy to learn to setup the equipment before the session. *

	1	2	3	4	5	6	7	8	9	10	
Strongly Disagree	○	○	○	○	○	○	○	○	○	○	Strongly Agree

I was able to setup the equipment and use the application after the first training session. *

	1	2	3	4	5	6	7	8	9	10	
Strongly Disagree	○	○	○	○	○	○	○	○	○	○	Strongly Agree

Efficiency

With the following questions we are trying to evaluate the level of attainable productivity of the user after they have learned the system

I was able to setup the equipment at home without wasting much time. *

	1	2	3	4	5	6	7	8	9	10	
Strongly Disagree	○	○	○	○	○	○	○	○	○	○	Strongly Agree

I was able to progress through the session using the application with ease. *

	1	2	3	4	5	6	7	8	9	10	
Strongly Disagree	○	○	○	○	○	○	○	○	○	○	Strongly Agree

Memorability (Not Applicable in this study)

With the following questions we are trying to evaluate the ease of remembering the system so that the casual user can return to the system after a period of time of non-use, without needing to learn again.

Few Errors

With the following questions we are trying to evaluate the capability of the system to support users in making less error during the use of the system, and in case they make errors, to let them easily recover

The error messages displayed by the application were clear and helped me fix the problem *

	1	2	3	4	5	6	7	8	9	10	
Strongly Disagree	○	○	○	○	○	○	○	○	○	○	Strongly Agree

I had no problem feeling stimulation on my ear for every session *

	1	2	3	4	5	6	7	8	9	10	
Strongly Disagree	○	○	○	○	○	○	○	○	○	○	Strongly Agree

Satisfaction

Measure of how pleasant the design is to use

I felt comfortable using the application *

	1	2	3	4	5	6	7	8	9	10	
Strongly Disagree	○	○	○	○	○	○	○	○	○	○	Strongly Agree

I felt comfortable setting up equipment *

	1	2	3	4	5	6	7	8	9	10	
Strongly Disagree	○	○	○	○	○	○	○	○	○	○	Strongly Agree

I felt comfortable wearing the sensors for the entire session *

	1	2	3	4	5	6	7	8	9	10	
Strongly Disagree	○	○	○	○	○	○	○	○	○	○	Strongly Agree

What did you do during the 20 minutes of electrical stimulation? *

- ☐ Close eyes and relax
- ☐ Watch TV or content on Netflix, Prime, Disney, etc.
- ☐ Use you phone to watch content or browse social media
- ☐ Listen to music
- ☐ Other:

Your overall feedback *

Your answer

Any suggestions for improvement

Your answer

Participant ID *

Your answer

Submit

Clear form

Never submit passwords through Google Forms.

This content is neither created nor endorsed by Google. - Contact form owner - Terms of Service - Privacy Policy

Does this form look suspicious? Report

Google Forms
